# Supplementary material for: Negative differential electrical resistance of a rotational organic nanomotor
Source: Beilstein J Nanotechnol. 2015 Dec 8;6:2332–7. doi: 10.3762/bjnano.6.240 (PMC4685900; doi:10.3762/bjnano.6.240)
Supplement: File 1 — Additional theoretical information. [file Beilstein_J_Nanotechnol-06-2332-s001.pdf]

Supporting information
for

Negative differential electrical resistance of a rotational organic nanomotor

Hatef Sadeghi, Sara Sangtarash, Qusiy Al-Galiby, Rachel Sparks, Steven Bailey* and Colin J. Lambert*

Address: Quantum Technology Centre, Department of Physics, Lancaster University, LA1 4YB Lancaster, UK

Email: Steven Bailey* - s.bailey@lancaster.ac.uk;

Colin J. Lambert* - c.lambert@lancaster.ac.uk

* Corresponding author

Additional theoretical information

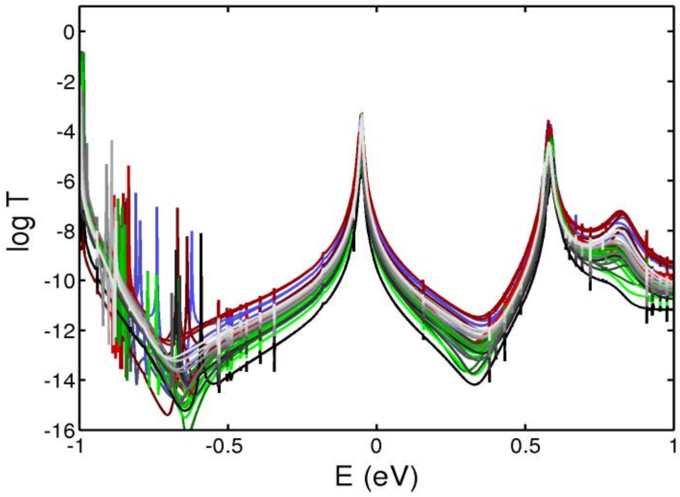

Transmission coefficient for full rotation of the rotor

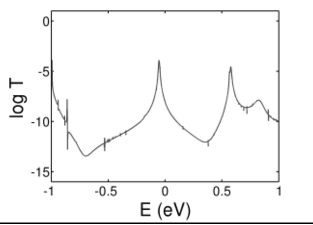

θ = 10

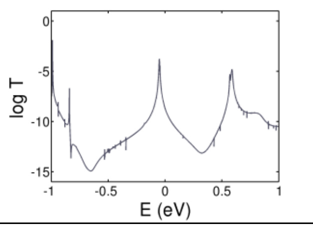

θ = 20

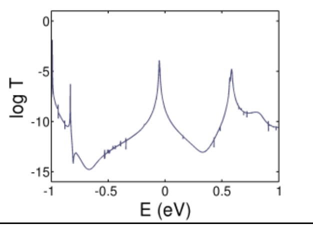

θ = 30

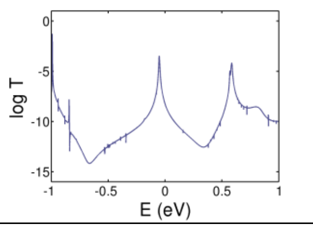

θ = 40

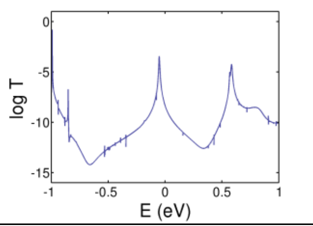

θ = 50

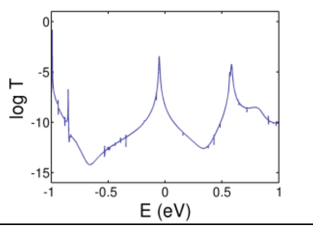

θ = 60

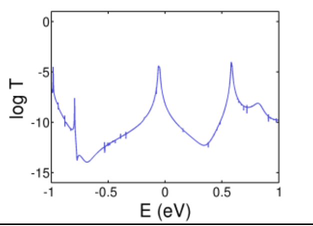

θ = 70

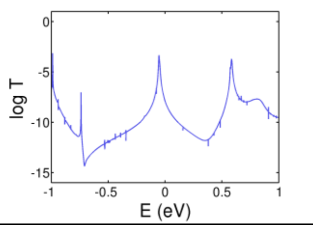

θ = 80

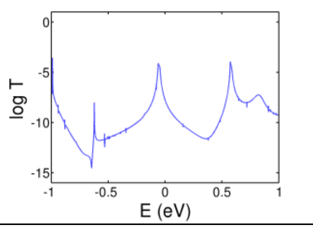

θ = 90

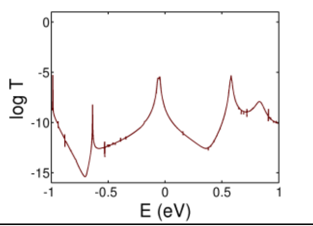

θ = 100

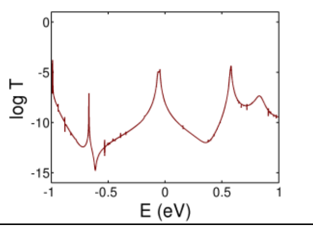

θ = 110

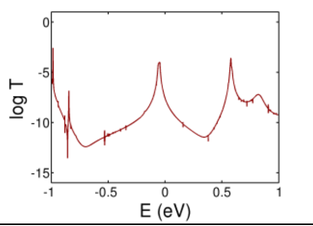

θ = 120

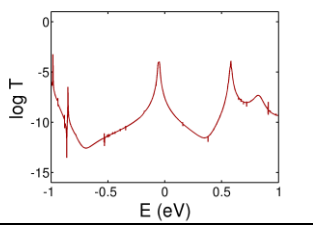

θ = 130

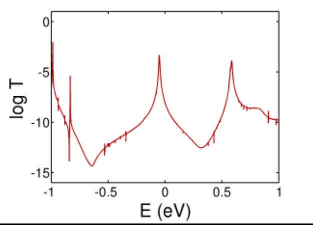

θ = 140

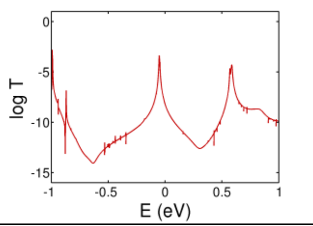

θ = 150

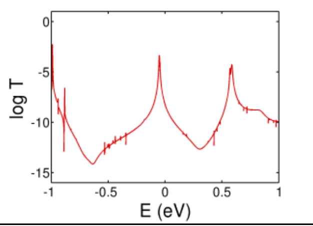

θ = 160

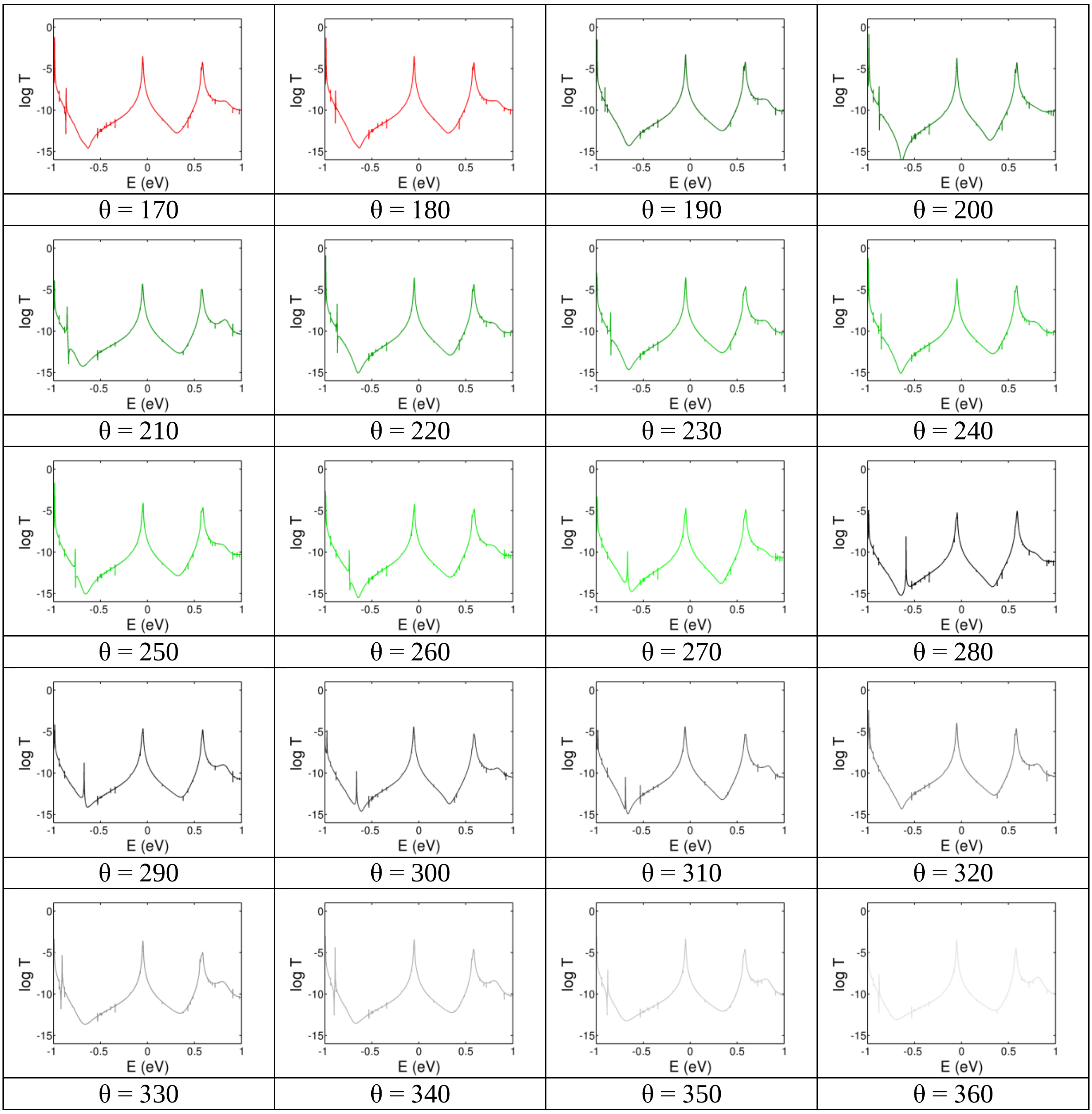

Figure S1: Transmission coefficient $T(E)$ of electrons with energy E passing from one fullerene to another for full rotation of the rotor.

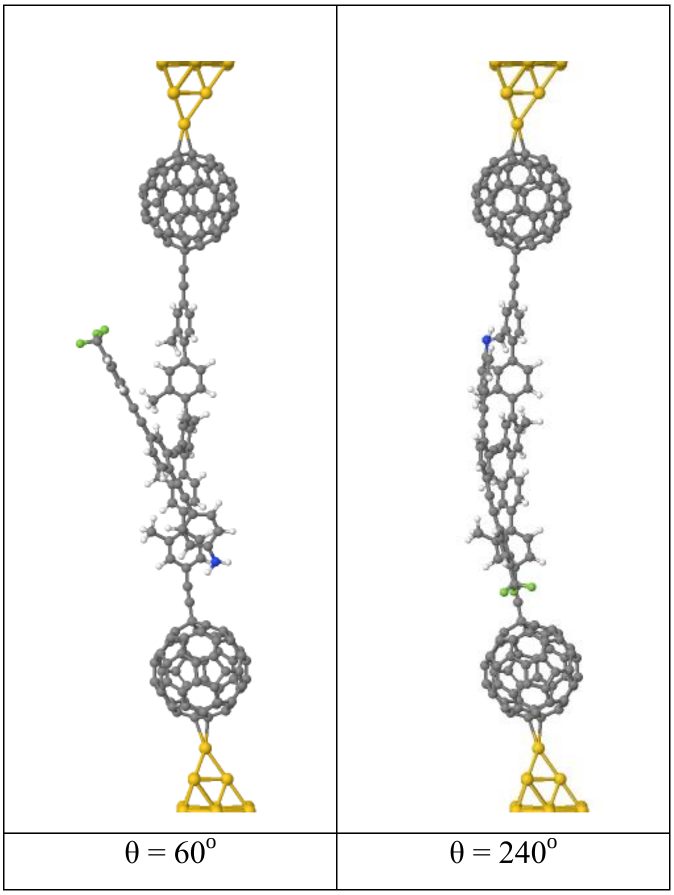

$\theta = 60^{o}$	$\theta = 240^{o}$

Figure S2: The molecular conformations corresponding to 60 and 240 degrees. Note that the pendant group is first rotated by θ and then the structure is relaxed. During geometry relaxation, the pendant group relax to the ground state which slightly changes the rotation angle θ.

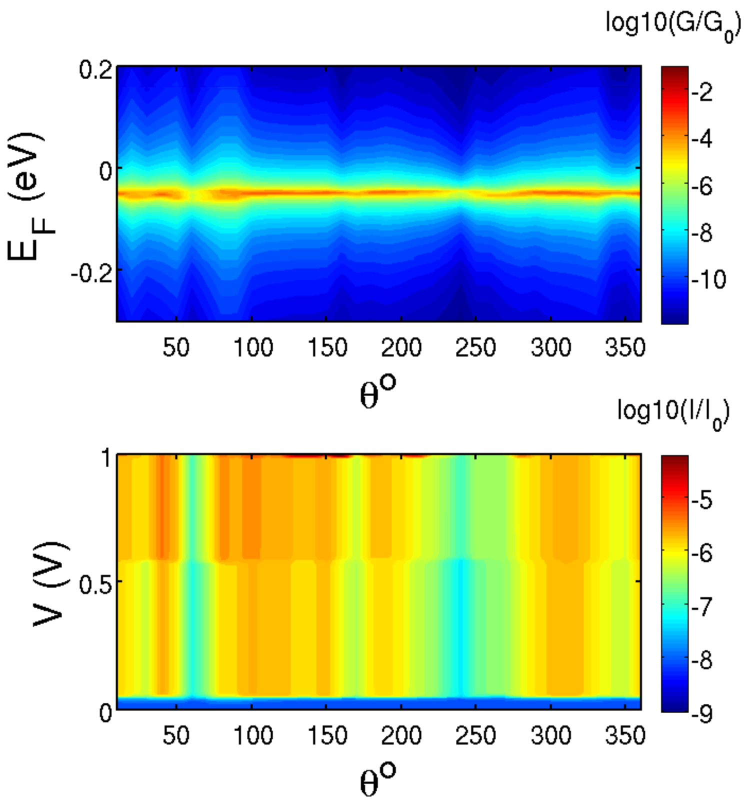

Figure S3: The contour plot of the conductance G/G_0 and current I/I_0 through the dumbbell molecule between two gold electrodes against angle θ and E_F (eV) at zero temperature.

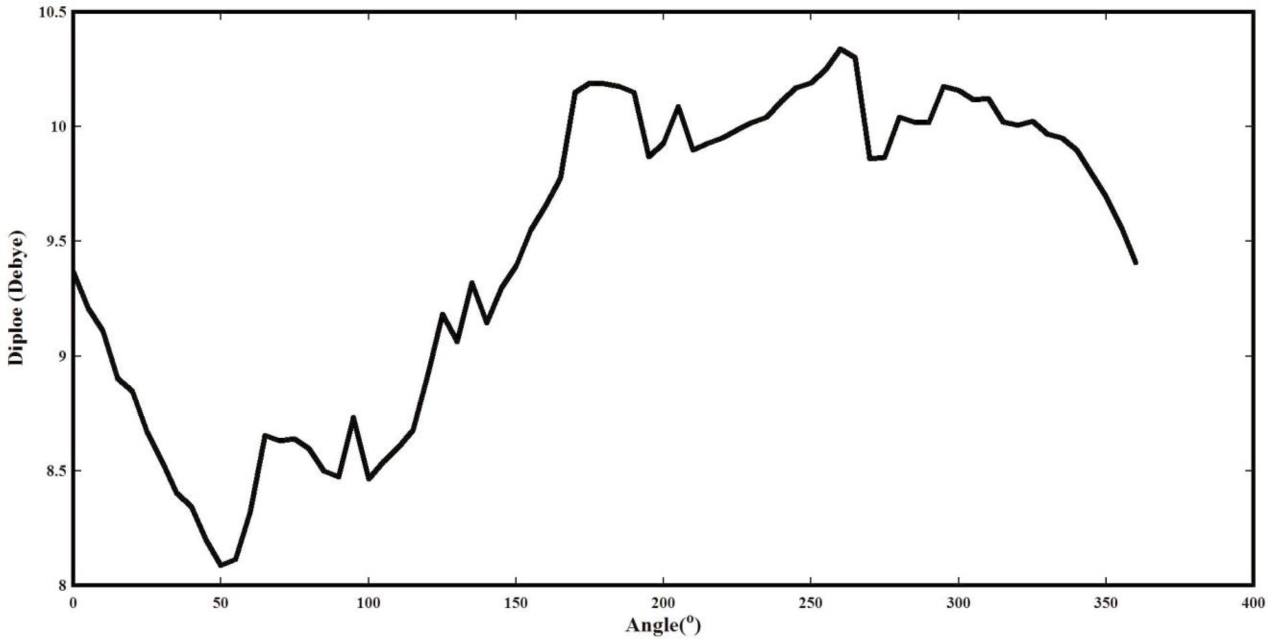

Figure S4: The dipole moment for the combined pendant group and backbone for all the rotation angles of the pendant group relative to the backbone is shown. The dipole moment shows oscillatory behaviour with a minimum at ≈50° of ≈8.1 debye and a maximum at ≈240° of ≈10.2 debye with an average of ≈9.4 debye. The length of the rotor and backbone is shown in Figure S5 to be 28.23 Å.

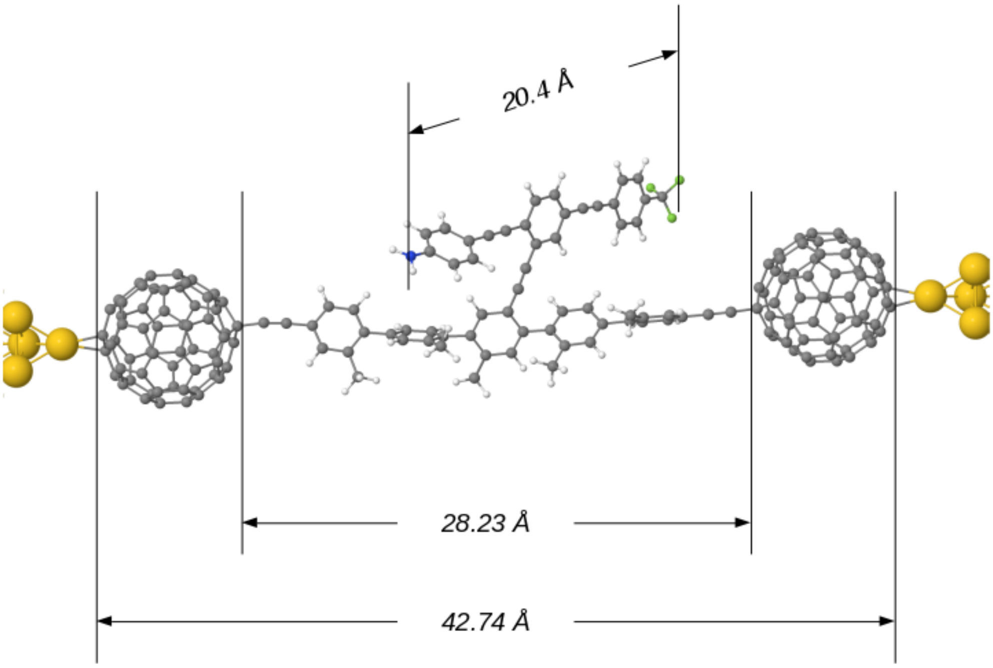

Figure S5: The length scales of the nanomotor.
